# Integrating Cognitive Behavioural and Islamic Principles in Psychology and Psychotherapy: A Narrative Review

**DOI:** 10.1007/s10943-022-01576-8

**Published:** 2022-05-19

**Authors:** Angie Cucchi

**Affiliations:** London Metropolitan University, London, UK

**Keywords:** Cognitive behavioural therapy, Integration, Islam, Cross-cultural, Psychotherapy

## Abstract

Standardisation of knowledge has become a by-product of globalisation, and western-based models are often seen as the ultimate answer to expertise and development. In light of this, some professionals have debated the feasibility of employing cognitive behavioural therapy (CBT) with Muslim communities. Debates have focused on CBT’s secular roots and its compatibility with a world where Islam permeates most aspects of life. This article highlights some of the theoretical dilemmas of integration and suggests ways to bridge the existing gap between secular and Islamic literature and avoid alienating those individuals who might feel uncomfortable with secular CBT teachings.

## Introduction

Contact, exchanges and reciprocal influences between different cultures are phenomena that date back to the origins of time, and indeed, history books are replete with narratives of encounters, clashes and movements between kingdoms and their inhabitants. However, whilst migrations—whether physical or cultural—are not a novel occurrence, the pace of the associated changes is. The advances in technology and transportation of last few decades have ensured that the opportunities for and the impact of these contacts have never been more immediate and far-reaching, creating a “global village” (McLuhan, 1989). As the world has become smaller and geographical, political and social boundaries have blurred, “globalisation” has created an unprecedented interdependency leading, on the one hand, to many positive changes and opportunities, whilst simultaneously bringing loss.

Many complain of the devastating impact of globalisation on indigenous traditions, customs, languages and values (Marsella, 2012). For example, Jensen and Arnett (2012) argue that migrations have contributed to the loss of traditional intergenerational systems of cultural transmissions. A similar complaint arises from Bristol-Rhys’ discussions with older Emirati women (2010), who lamented the loss of a sense of community and identity following the rapid globalisation of their country. Relatedly, Thomas (2013) describes how most tertiary education systems in the Gulf have adopted western models of teaching and curricula, use English as the language of instruction and have replaced regionally educated faculty members with western-educated ones, with significant impact on the region’s sense of identity and native language.

Given that the process of globalisation is driven by a few nations (Marsella, 2005), it tends to reinforce adverse power dynamics in which one party is seen as needing to be “modernised” according to preconceived standards. Marsella (2005) describes this form of globalisation as “hegemonic” and regards it as a legacy of colonialism. Whilst the nineteenth-century colonialist mentality prompted the unequivocal and often forceful replacement of traditional systems, hegemonic globalisation entails a much more subtle and less overtly intrusive alteration, often achieved through exporting education and health systems (Fernando, 2014; Watters, 2010).

Against this background, the discourse around globalisation has more recently emerged concerning mental health. Although indigenous models for the aetiology, nature and treatment of mental illness have been present throughout history, mental health, psychology and psychotherapy have traditionally been western-dominated (Fernando, 2014). Furthermore, owing to the current global power imbalances, any change tends to occur in line with a westernisation of the non-western (Fernando, 2014). As a result, concepts, hypotheses, models and even the phenomenology of mental illness have been standardised to a point of almost complete dominance of Eurocentric and North American models and perspectives.

In light of an increased sensitivity towards the role that psychology played in contributing to and perpetuating a “hegemonic science”, hierarchies of human values and systemic inequalities, the profession has more recently made a commitment towards acknowledging the limitations of western-based clinical frameworks and honouring other healing approaches that have roots in Indigenous and other non-western cultural traditions (APA, 2021). The present paper positions itself at the heart of this discourse and aims to explore some of the key considerations of the intersection between globalisation, cultural diversity, mental health and psychology/psychotherapy.

## Psychology and Psychotherapy’s Dominant Discourse

During the last five decades, awareness of mental health issues has consistently increased across the globe, together with a reported rising incidence of mental health difficulties (Eloul et al., 2009; Nasir & Abdul-Haq, 2008; Obermeyer et al., 2015; WHO, 2021). This trend has been particularly salient during the COVID-19 pandemic, which saw a further steep surge in severe psychiatric and psychological presentations (Nochaiwong et al., 2021) either as a direct effect of COVID-19 or as a result of financial and economic issues related to economic lockdowns (World Bank, 2020).

In addition, the socio-economic turmoil in some developing countries has triggered the flow of refugees to high-income countries and many of these migrants tend to have unmet psychological needs. As a result, the quality of mental health care is a timely issue for practitioners, researchers and policymakers (Lim et al., 2018).

Whether in an attempt to create, or improve services, or in the aftermaths of natural disasters, or social violence, western-educated experts are routinely flown across the globe to conceptualise, set up and run psychological and psychotherapeutic services. Even in their homeland, these professionals routinely work with individuals whose background may be completely different from their own. Many of these professionals have little knowledge of indigenous cultural backgrounds and rely on western conceptualisations and principles which are often at odds with many non-western cultures that do not use existing biomedical and western psychotherapeutic frameworks (Fernando, 2014). In particular, psychologies in non-western cultures are often embedded in spiritual, religious and philosophical paradigms (Fernando, 2014), which are not captured by secular, dualistic frameworks.

Explanatory models for the aetiology of distress, as well as the idiom of distress itself, tend to be very different in western and non-western cultures, with the latter at times relying on explicative models rooted in “sihr” (sorcery), evil eyes and “jinns” (spirits; Lim et al., 2018). The idiom of distress is also different, with non-western societies adopting more somatic metaphors than psychological ones (Al-Adawi et al., 2002a, 2002b). Given that western-trained health professionals tend to use explanatory models that exclude such cultural–religious concepts, individuals may feel misrepresented (El-Islam & Dagga, 1992). Conceptualising the source of one’s own affliction as rooted in the supernatural forces, rather than on biopsychosocial elements (Engel, 1980) naturally impacts on help-seeking behaviours, as well as engagement with different services. Taking into consideration more holistic frameworks is, then, crucial for psychology and psychotherapy not to alienate themselves from the very same people they wish to serve.

Given the popularity of cognitive behavioural therapy (CBT) in the West, and its short-term, protocol-led and highly manualised nature which greatly appeals to training institutions, some professionals have called for exporting this approach to working with Muslims (Al Sharbati et al., 2014; Hodge, 2008; Hodge & Nadir, 2008). Nevertheless, the feasibility of applying secular, western-based models to cultures whose foundations are intrinsically shaped and connected to Islam has been questioned (Al-Abdul-Jabbar & Al-Issa, 2000; Bentall, 2003; Fernando, 2014). In fact, Islam can pervade nearly all aspects of life, from food choice, to daily routines, social interactions, education, architecture and health care (Haque, 2004a; Hickey et al., 2016). Failing to consider this would arguably alienate a segment of the population that might fear not having their beliefs system represented, or even understood. Indeed, many of the fundamental principles of the dominant discourse within contemporary secular psychology and psychotherapy are intrinsically opposite to those in the Islamic narrative (Badri, 2008) and for some people, engaging with secular, western-based approaches might even be perceived as a *threat to parts of Islam itself* (Asamari, 2018, p.58).

This threat stems from the traditional reluctance of Western psychology and psychotherapy to engage dialogues around institutional theology. Freud’s (1976) idea that religion is a form of illusion to work through and the aspirations of the early psychoanalysts to create a therapeutic framework based on the nineteenth-century physical sciences (Rudden, 2004) have arguably contributed to the suspicion that the Islamic tradition has historically harboured towards western psychological and psychotherapeutic approaches. In fact, like the founder of psychoanalysis, the majority of early psychoanalysts were not religious—often openly anti-religious in fact (Issroff, 1999)—and neglected perspectives that viewed theology as worthy of psychoanalytic exploration (Budd et al., 2005).

A similarly rejecting stance towards religious beliefs was adopted by Rogers (1951), who renounced institutional and legalistic teleological orthodoxy as stifling humankind’s growth (Jones & Butman, 1991). Rogers (1951) argued that religions imposed critical conditions of worth that are antithetical to the basis of the person-centred approach. Although he remained connected to the mystical side of being, religious dogma such as those embraced by Islam was rejected in favour of spiritual connectedness (Fuller, 1982). Meaning was to be sought and found within personal experience, not outside of it, prompting some to describe the approach as setting the base for the erosion of American religiosity (Browning, 1980; Coward, 1989; Gross, 1978; Jacoby, 1975) and the emergence of the modern self-indulgence, self-aggrandisement (Bergin, 1980) and narcissism (Watson et al., 1984) that many religions lament.

CBT’s early pioneer Albert Ellis (1980) similarly attacked teleology and its practices, which he also argued were in opposition to mental health goals. Like Rogers (1951), Ellis (1980) argued that the absolutistic and perfectionist standards imposed by religious dogma promoted self-critical thoughts when such standards were not met. Ellis believed this process eventually led to the most corroding human emotions: anxiety and hostility. Although his view of religion softened over the years, his position remained that dogmatic devotion to religious creeds created emotional disturbances.

A comparably discrediting stance towards theological beliefs has been adopted by existential psychotherapists, who argue that faith emerges from human beings’ attempts to soothe death and existential anxieties and find a purpose in an intrinsically nonsensical world (Yalom, 2002). Yalom (2002), on whom many of the tenets of existential psychotherapy rest upon, advocates that humankind’s main life task is to invent a purpose solid enough to withstand the nothingness of life. Religion is believed to achieve this function by suggesting meaning and rituals and by providing a sense of belonging. It follows that *we create Gods for our comfort* (Yalom, 2002, p. 308), rather than us being created by God, as Islam professes. Other philosophers whose writings also form the pillars of existential psychotherapy, took a similar stance and claimed that God is dead (Nietzsche, 1974), that God is non-existent (Sartre, 1943) and that religion is a glow-worm that is only visible in the darkness (Schopenauer, 1851), hence widening the gap between religion and therapy further and contributing to the weariness that Islam harboured towards psychotherapy.

Some psychotherapists took a more benevolent and, at times, embracing stance towards religions, valuing it as part of a meaning-making process (Frankl, 1962), a broader understanding of what it means to be human (Gantt & Melling, 2009), or a mystic experience linked to a collective unconscious (Jung, 1938). In addition, the field of western psychology and psychotherapy has more recently been pervaded by an increasing interest in spirituality and mystical experiences (Black, 2006; Epstein, 2007). Even though the therapeutic space has become an environment where the exploration of mysticism is welcome and valued, psychology and psychotherapy ultimately embrace a pluralistic attitude that directly contradicts Islam’s ontology and the claims of exclusiveness and “Truth” professed by institutional teleological orthodoxy. Furthermore, psychological and psychotherapeutic frameworks explain the complexity of human experiences according to horizontal causality. This concept is at the expense of the vertical, metaphysical causality framework embraced by the Islamic doctrine (Kaplick et al., 2019).

### The Implications of the Dominant Discourse

The above arguments are outlined by several authors (Haque, 2004a; Kaplick et al., 2019; Weatherhead & Daiche, 2010) and beautifully captured by one of Rothman and Coyle’s (2020, p.22) participants, who states that “*a Western psychology perspective doesn’t appreciate a wide understanding of the self* or *(…) the possibility of some external influence on the person in terms of (…) unseen supernatural beings”.* Ultimately, when modern social sciences separated from philosophy and established themselves as a scientific discipline, they emphasised the secularisation of knowledge. Social sciences embraced, instead, observation and experimentation at the expense of divine scriptures and metaphysical elements, whose roles in explaining behaviour were completely rejected (Haque, 2004b). However, this stance is in direct contradiction with the Islamic view which instead embraces and accepts these factors and views scientific development as based on faith (Haque, 2004b).

These substantial ontological and epistemological differences, and the legacy of psychotherapy’s earlier stance towards religious beliefs have had profound ramifications. It is reported that Muslims tend to be more ambivalent towards approaching mental health services than other religious groups (Hedayat-Dib, 2000; Pilkington et al., 2012; Sheikh & Furnham, 2000). *Whatever happens is from Allah, and so a creation of Allah cannot help; we’ve never been told about the quality of what’s inside (mental health services), so we do not know; you don’t need that (a psychologist) because you are talking to friends and family* say Weatherhead and Daiche’s (2010, p.81, p.84) participants, emphasising the reluctance to rely on mental health services.

Whilst it is arguable that Muslims’ ethnic and national backgrounds are incredibly diverse, that the Islamic theology is not homogenous and that the multiplicity of discourses and practices within Islam are shaped by historical and political movements (Eickelman & Salvatore, 2006), it has also been pointed out that Muslims across the globe usually endorse the same worldview based on the Quran and the Sunnah (Haque, 2004a). It has also been pointed out that, even though there are cultural and individual variations in some of the beliefs and practices related to Islam, a strong connection to religion remains—*we are not good Muslims, but during times of distress, the first thing that comes to mind is God* (Weatherhead & Daiche, 2010, p. 80). Haque and Keshavarzi (2014, p. 298) describe Islam as a “*unifying cultural element*” despite in-group variability, and Sanadjian (1997) describes a homogeneity of faith and heterogeneity of practice.

The strong connection many Muslims have to Islam also translates in more Muslims than other religious groups resorting to religion in response to stress (Adam & Ward, 2016; Bhui et al., 2008; Cinnirella & Loewenthal, 1999). For example, a recent survey of American Muslims suggests that only 11% would seek therapy compared to 21% who would seek help from a family member and 19% who would approach religious leaders (Aloud & Rathur, 2009). The percentage of people who would opt for traditional healers rather than mental health professionals is much higher in the Greater Middle East, with 60% of Omanis, 58.5% of Iraqis, 42% of Saudis and half of the people surveyed in the UAE confirming that they had consulted religious healers (Al Hemiary et al., 2014; Al Rowais et al., 2010; Okasha, 2004; Salem et al., 2009). Interestingly, Salem et al (2009) found that many patients continued to seek treatment from faith healers even after engaging with mental health services.

Given that many Muslims attribute the causes of mental illness to the supernatural (Al-Solaim & Loewenthal, 2011; Salem et al., 2009), or a test from God, or indeed to being disconnected from the Creator (Ghazali, 1986; Rothman & Coyle, 2018), and that many believe that recovery ultimately comes from God, these findings should not come as a surprise. It follows that many Muslims continue to feel uncomfortable in seeking psychological support for fear that this might conflict with or not consider their religious beliefs (Sabry & Vohra, 2013). The findings suggest that unless psychotherapeutic approaches consider and incorporate indigenous values and beliefs, psychology and psychotherapy may continue to be seen by some Muslims as an alien discipline, if not hostile (Weatherhead & Daiches, 2010).

### The Dilemma: Integration or Non-integration?

**Fig. 1:**
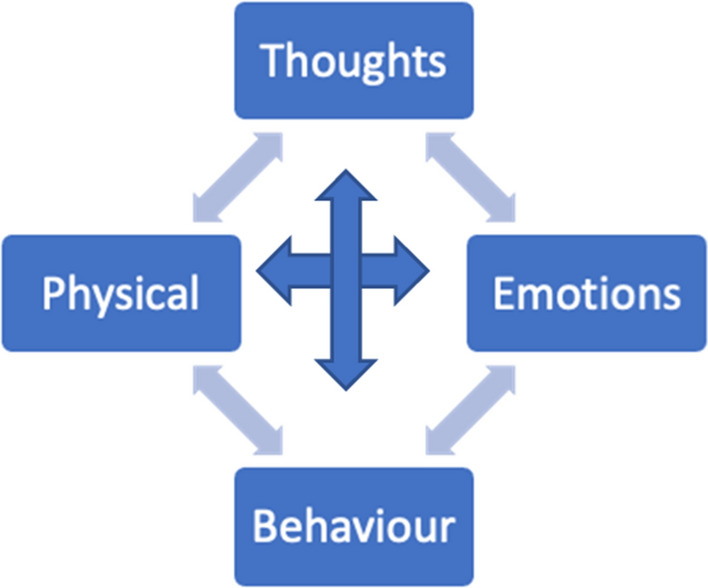
Hot Cross Bun (Greenberg & Padesky, 1995)

**Fig. 2:**
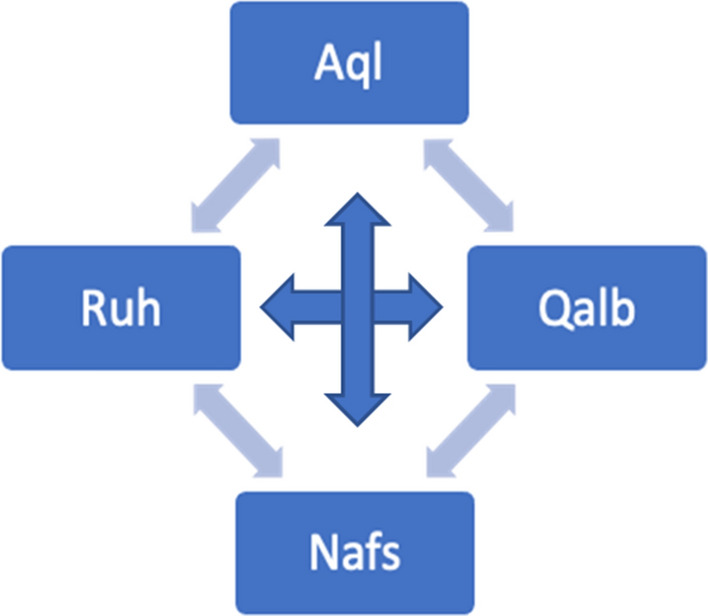
Ghazali’s (1986) conceptualization of the human psyche

CBT is grounded in the secular tradition, the Cartesian dualism and it is not value-neutral. Its principles are rooted in the American’s value system and its emphasis on cognition, logic and rational thinking stems from and reinforces dominant cultural discourses, including definitions of rationality (Kantrowitz & Ballou, 1992) that easily disregard spirituality. Historically CBT has shown little to no attention to religion (Imawasa & Hays, 2018). At the same time, some clinicians have argued that the model is nevertheless more compatible with Islamic values than other approaches (Sheik, 2018; Thomas, 2013). It has been suggested that the CBT’s principle of interdependence between cognitions, thoughts, physical sensations and behaviour (Greenberger & Padesky, 1995—Fig. 1) is consonant with Ghazali’s (1986) conceptualisation of the human psyche (Fig. 2).

Ghazali (1986), an eleventh-century Islamic scholar, suggested that human nature comprises of four interconnected elements: the “*aql*” (intellect), the “*qalb*” (heart), the “*nafs*” (self) and the “*ruh*” (spirit). The “*aql*” is believed to be the logical part of human beings, that part that is mostly concerned with rationality and logical thinking (comparable to CBT’s cognitions) whereas the “*qalb*” is believed to be that part of human beings where all emotions reside. It is noteworthy that Ghazali (1986) did not refer to, nor attempted to engage with the “heart” in a biological sense (Kemahli, 2017). Instead, Ghazali’s (1986) conceptualisation of the “heart” was more spiritual, psychological, and was used to describe the most important faculty in pursuing knowledge and comprehending the subtleties of the tangible world (Çağrıcı, 2013).

Whilst the role of the “*aql*” was believed to be that of filtering out maladaptive thoughts and unhelpful behavioural inclinations, the “*qalb*” was believed to perform and oversee regulatory functions for all the elements of the psyche (Haque, 2004a; Kemahli, 2017). Acknowledging the interconnected and interdependent nature of the “*qalb*” and the “*aql*”, Ghazali (trans. 2000, p.18) warned that: “*The heart is like the sultan of a city (…); and one’s faculty of reason, endowed with knowledge and will, is the sultan’s vizier*”. Just like the vizier manages the affairs on behalf of the sultan, who remains the highest authority, the “*qalb*” was believed to acquire a central role in maintaining equilibrium in and between all the elements of the psyche. This conceptualisation contrasts with CBT’s model of human nature that, instead, views thoughts in the driving seat.

Therapists are reminded that unless the “*qalb*” (the emotions), is addressed in therapy, real change won’t happen as the “*aql*” alone is believed not to be sufficient to initiate long-lasting healing (Rothman, 2018). In line with what argued in the Islamic tradition, CBT’s clients often complain of a dissociation between a rational belief (a cognition) and “the way it feels” (the emotional response), a phenomenon known in CBT as the “heart–mind lag” (Lee, 2005). Clients might intellectually know that they are loved, worthy or valued, yet they might have a deep and engrained feeling of not being. Addressing this “lag” between cognition and the visceral feeling is crucial and a key component of CBT for change to occur (Kennerley et al., 2017), just as Muslim scholars claimed.

The importance of the *“qalb”* in the Islamic model is further exemplified by the role given to the latter in establishing adaptive/maladaptive patterns with the other elements of the model. For example, the “*qalb*” can either turn towards the “*nafs*” potentially triggering a maladaptive cycle and/or can elevate itself to a higher state by turning to the most spiritual element of the psyche, the “*ruh*” (Rothman & Coyle, 2020). When the “*qalb*” turns towards the “*nasf*”, it falls prey to the *nafs*’ most instinctual and primitive impulses.

An exploration of Ghazali’s (1986) conceptualisation of the “*nafs*” suggests that the latter can be associated with the CBT’s behavioural component of the Hot Cross Bun and, particularly, with those safety behaviours and behavioural inclinations that although not helpful, are performed to soothe an immediate urge. Unable to tolerate the dissonance between the “*aql*” and/or “*qalb*”, an individual might surrender to behavioural impulses to silence those components of the psyche. Indulging the “*nafs*” might produce some desperately sought relief, albeit short term and ephemeral. Nevertheless, this relief might trigger a vicious cycle in which anxiety is experienced if the “nafs” is not constantly indulged. As a result, the lack of harmonious equilibrium between the different elements that constitute the human nature is maintained, as outlined in the Greenberg and Padesky’s (1995) model.

Should instead, the “*galb*” turn towards the “*ruh*”, it would head towards the purest part of the psyche. Almost antithetical to the “*nafs*”, the “*ruh*” is the most metaphysical part of the human nature, that part that strives to connect to the divine. Believed to have been bestowed upon humans by God—“*I have blown My spirit into it*" (Holy Quran, 2001, Al Hij’r,15:29)—the “*ruh*” allows human beings to claim a greater affinity to the spiritual than any other living creature. The “*ruh*” has been described as the pure and unshaken segment of the psyche where God’s imprint resides, that part of human nature where individuals can find divine knowledge and healing (Rothman & Coyle, 2018). Given the intrinsic divine nature and origins of the “*ruh*”, whose natural inclination is believed to be seeking closeness to God (Lodi, 2018), human beings are understood to be naturally geared towards seeking spirituality and closeness to the Divine.

Missing from the cognitive behavioural framework, the concept of the *“ruh”* resonates better with the humanistic principle of “self-transcendence”, the ultimate stage of the “self-actualisation” process (Maslow, 1962). Regarded as “*the very highest and most inclusive or holistic levels of human consciousness, behaving and relating (…) to oneself, to significant others, to human beings in general, to other species, to nature, and to the cosmos* (Maslow, 1971, p. 269)*,* self-transcendence is the stage whereby a person moves beyond their boundaries as an individual and connects with the spiritual all human beings share. Similarly, when human beings transcend the behavioural inclinations dictated by the “*nafs*” and, instead, cultivate the mystical nature of the “*ruh*”, they achieve optimal level of functioning and well-being.

Regardless of the theoretical speculations about which Islamic component matches (if any) its western counterpart, these elements are believed to be intrinsically connected (Ghazali, 1986), so that imbalance in one necessarily precipitates changes in the whole system. For example, the lack of a sound logic (*aql*) might impair judgments related to the pursue of the metaphysical and spiritual (*ruh*) against instinctual impulses (*nafs*). This, in turn, might impact on a person’s emotional core (*qalb*). On the contrary, a sound intellect, which works in unison with a healthy emotional regulatory system would not struggle to contain lower behavioural inclinations, knowing that its role is to transcend those to reach a spiritual connection with the divine. A hadith from Al Bukhari (translation by Khan, 1998) confirms these interconnections: “*there is an organ in the body, that if it’s pure, the whole body is pure and it is diseased, the whole body is diseased: the heart*”.

Notwithstanding the different emphasis between this position, which claims the heart to be the “command centre” (Lodi, 2018) and CBT, which accentuates the intellect, both models rely heavily on the interconnectedness between each element of the cycle. In addition, the above hadith from Al Bukhari arguably introduced the fourth segment from the “Hot Cross Bun”: the body, the physical realm. Although plausibly used loosely in this context, the term “body” reminds us that Islamic scholars also viewed the physical sphere as inextricably interconnected to the other parts of the human psyche. Ghazali’s view (1986) also confirms that it’s inconceivable to think that the spiritual inclinations of the heart can be accessed without taking care of the physical body (Kemahli, 2017).

In addition to the similarities between the two models as described above, the supporters of an integration of CBT and Islamic principles argue that the scientific nature of CBT aligns well with the importance that Islam places on science and a scientific exploration (Hodge & Nadir, 2008). Ashy (1999) emphasises the importance that the Quran places on logical thinking and highlights that the Holy Book uses rational and analytical arguments of persuasion of its Truth. It can be claimed that this process is arguably in line with several CBT cognitive techniques that require clients to pursue logical arguments, to gather evidence pro/against and to challenge thoughts. In addition, Thomas (2013) equates the CBT evidence-based practice of challenging negative automatic thoughts (NATs) to the Islamic concept of “Husn al-Dhann” (positive regard, having a good opinion). The meaning of “Husn Al-Dhann” is to encourage people to avoid making assumptions and to retain a balanced and realistic view. This Islamic practice, just like the secular CBT one, is believed to have a positive influence on individuals’ well-being.

To further support the claim that CBT principles are in line with Islamic thinking, Rothman (2018) suggests that cognitive restructuring—a pillar of CBT—is in line with the teachings of Al Balkhi (Badri, 2013), a ninth-century Muslim scholar who advocated for the importance of restructuring cognitions in line with religious teachings. Whilst Al Balkhi is believed to be one of the first to advocate for the role of psychotherapy to manage mood—“*gentle encouraging talk that brings back some happiness*” (Badri, 2013)—he is certainly not the only one, and other early Muslim scholars are also believed to have been pioneers in the development of an early form of an Islam-congruent “cognitive therapy”.

For example, Al Kindi (Haque, 2004b) is credited for arguing that sorrow is not within us but rather brought upon us by ourselves and that cognitive strategies ought to be used to tackle depressive states. This statement is a clear echo of CBT’s Stoic philosophical foundation that suggests that “*men are disturbed not by the things which happen, but by their opinions about the things*” [(Epictetus, (125 C.E.) 1991). Similarly, At-tabari (Haque, 2004b) suggested using “wise counselling” to make patients feel better and several other early Muslim scholars advocated for the use of thought-evaluating and thought processing interventions similar to the ones described by Thomas (2013) and Rothman (2018) to tackle psycho-spiritual ailments (see Haque, 2004b for a full review).

It is also arguable that the psychoeducational element, the more direct nature of CBT compared to other Western modalities and the stance of the CBT therapist as a teacher/coach/scientist (Kennerly et al., 2011) might be more consonant with the expectations of some Muslims coming from the Arab and South Asian cultures (Haque, 2004b). In line with the CBT therapeutic stance, Al Issa (2000) equates the dynamics between Muslim therapists and their clients to the ones of teachers and learners. He mentions that Muslim clinicians need to be more directive and assertive than other western therapists as Arab and South Asian cultures may expect and value more expert advice and self-disclosure than western cultures.

Whilst there seem to be several arguments in favour of the application of traditional CBT to Muslim communities, there are also many arguments against it. First of all, Islamic principles lay on ontological absolutism, which is in stark opposition to the constructive nature of CBT. Islam maintains that there is an absolute Truth and that “should” ought to exist (Sheikh, 2018). Instead, CBT shies away from “should” (Beshai et al., 2013) and has ontological and epistemological foundations on constructivism. Moreover, CBT’s reliance on an internal locus of control and self-determination seems to be in complete opposition to Islam’s belief that “*Allah is the best of Planners*” (Holy Quran, Al Anfal 8:30) and that a believer ultimately has to understand and appreciate the divine plan, even though it might not be what the person had wished for (“*You may hate a thing and it is good for you; and you may love a thing and it is bad for you. And Allah knows, while you know not*”—Holy Quran, Al Baqarah, 2:216).

In addition, the self-determination concept, as well as CBT’s highly individualistic nature and focus, cast doubts on its application to a belief system that emphasises worship (“*I did not create the Jinn and mankind except to worship me*”—Holy Quran, Ad-Dhariyat 51:56). The associated goal can then be inherently different: self-determination for one versus rectification of Islamic psychological processing (Keshavarzi & Khan, 2018), closeness to God and development of the spiritual self for the other. And whilst the two might coincide, that is not a given.

Furthermore, CBT’s conceptualisation of the individual as thoughts, emotions, physiology and behaviour seems to be missing a crucial element of the Islamic framework: the soul. In fact, Islam’s formulation of the individual rests on the vision of integration between the intellect, the heart, the self, the body and the soul, connected to the external environment and to God. Tawhid, or unity, is the guiding principle in Islam, a principle upon which Islamic sciences and medicine also rest. Tawhid conceptualises the universe and the creatures that live in it as a dynamic yet unified system where all are interconnected. So, whilst the concept of interconnectedness is inherent to conceptualisations of difficulties outlined by CBT’s Hot Cross Bun, the latter omits a crucial aspect: the spiritual realm.

In other words, whereas CBT focuses on the mind and cognitions’ impact on individuals’ experiences, Islam engages the soul and the heart in conceptualising the “*self*” (Inayat, 2005; Keshavarzi & Haque, 2013). The heart, and not the mind, is believed to be the centre of human beings in the Islamic tradition. Although some professionals argue that the “*qalb*” ought to be the real focus for therapists (Lodi, 2018; Rothman, 2018), others emphasise that early Islamic scholars had also stressed the central role of cognitions on influencing the other elements of the psyche (Keshavarzi & Khan, 2018), as seen above. Ultimately, however, whether the heart or cognition is the focus, the aim is to purify the soul and achieve psycho-spiritual well-being in line with Islamic principles (Haque, 2004b).

Far from reaching more clarity on the topic, the above points encapsulate the complexity of perusing western-based approaches with Muslims’ communities, where Islam represents not only a religion, but a way of thinking and life. It is apparent that although some of the techniques and practices of CBT might be helpful and offer important tools to address psychological distress, its current ontological and theoretical underpinnings are not necessarily aligned with an Islamic discourse. It has been suggested that this mismatch might explain some Muslims’ reluctance to engage with secular approaches and associated psychotherapeutic services (Amri & Bemak, 2012; Killawi et al., 2014). To address these incongruencies, it has been argued that professionals ought to work both at a theoretical and practical level to (a) identify indigenous views on knowledge and redefine the subject of psychology/psychotherapy from an Islamically oriented perspective; (b) expand theoretical frameworks to incorporate indigenous conceptualisations of the psyche; and (c) convert secular interventions into more culturally appropriate ones (Haque, 2004b). Some attempts have been made (Al-Abdul-Jabbar & Al-Issa, 2000; Haque & Keshavarzi, 2014; Rothman & Coyle, 2020; Sabki et al., 2019).

### The Evidence Base

As discussed, western-based approaches are routinely used with Muslim communities worldwide. Furthermore, globalisation in the form of western influence has also shaped the practice and teaching of psychology and psychotherapy in predominantly Muslim countries like Saudi Arabia (El-Naggar, 2012), UAE (Al-Darmaki & Yaaqeib, 2015), Iraq (Kizilhan, 2020), Bangladesh, Pakistan (Blowers et al., 1987), Malaysia, Indonesia (Geerlings et al., 2014) and others predominantly Muslims African countries (Nwoye, 2015). Importing highly manualised and protocol-led interventions such as CBT might sound like the perfect solution for training institutions and service providers that wish to rely on evidence-based practice, yet lack regional accrediting and regulatory bodies. However, without adequate considerations on the required cultural adaptations, such models might be implemented too rigidly and insensitively, hence contributing to further suspicion towards mental health services and professionals.

In light of the above considerations, and in order to explore the feasibility of developing a culturally sensitive cognitive behavioural model in a predominantly Muslim country, Cucchi et al. (2020) devised an intervention study in Iraq. The aim was to investigate the potential effectiveness of the traditional CBT approach and which variables were associated with better outcomes to systematically build on those to develop a culturally sensitive model. The study had a small number of participants and so is best seen as a pilot-scale trial, and indeed, larger-scale trials are needed. Nevertheless, its importance is apparent as the study is the first of its kind. The results suggest that traditional CBT significantly reduced symptoms in a sample of patients diagnosed with Obsessive Compulsive Disorder (OCD). The longer the treatment, the better the improvements, substantiating the long-term impact of the intervention.

Although these results were promising, Cucchi et al. (2020) also reported that 39% of the participants were still symptomatic at the end of treatment. This finding, the reluctance of some segments of the population to engage with traditional psychotherapy, and the potential for dropout rates in similar larger-scale studies on non-western populations (Foa & Kozak, 1996; Volpato Cordioli et al., 2003) make devising more effective interventions a priority. Given that Cucchi et al. (2020) found that commitment towards the CBT approach was significantly correlated to positive outcomes, exploring ways in which commitment can further be fostered seems pivotal. As a result of the significance that Islam has in some people’s life, it seems important to investigate whether a spiritually modified version of CBT might increase commitment and engagement with therapy.

Spiritually modified CBT frameworks are slowly being introduced in the literature (Al-Abdul-Jabbar & Al-Issa, 2000; Haque & Keshavarzi, 2014; Pearce et al., 2015; Rothman & Coyle, 2020; Sabki et al., 2019). However, they currently lack a unifying theoretical framework and outcome research studies, including secular VS Islamic-congruent Randomised Controlled Trials (RCTs), are few. The rarely available evidence indicates that Islamically modified interventions produce significantly faster improvements in anxiety (Alagheband et al., 2019; Azhar et al., 1994; Razali et al., 2002) and depressive symptoms (Alagheband et al., 2019; Azhar & Varma, 1995), although the gap between secular and religious interventions narrowed after six months. Furthermore, the literature also suggests that Quran recitation alone can produce significantly greater results than secular interventions in tackling symptoms of depression in a cohort of Muslim women (Rafique et al., 2019. Given the scarce but promising evidence base, an effort to understanding human experiences from Islamic frameworks ought to take precedence.

### “Integration or not?” The Way Forward

**Fig. 3:**
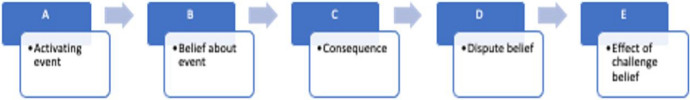
ABCDE model (Ellis, 1962)

Whilst therapists and researchers alike need to be mindful not to assume homogeneity of adherence to spiritual and religious practices, several professionals suggested incorporating the client’s religious beliefs into the original secular cognitive model (Hamdan, 2008; Lodi, 2018; Pearce et al., 2015; Rothman, 2018). Building on Ellis’ (1962) “ABCDE” model of Rational Emotive Therapy (Fig. 3), Pearce et al. (2015) suggested the ABCD-R-E model. Albeit non-specific to Islam, the manualised framework offers an interesting start from which to incorporate more specific Islamic beliefs.

Whilst the A stands for the activating event, B for the belief, C for the consequence and the D for the dispute of the belief, the R incorporates the religious creed that is missing from the secular model. Questions like “how does your view of God/religion help you to challenge the negative thought/suggest alternative beliefs” can offer a more holistic view and personally meaningful reflection for some clients. This, according to Pearce et al. (2015), provides the appropriate basis from which to reach an effective new belief and consequence (E). Pearce et al. (2015) recommend a 10-session format in which elements of psychoeducation to the model, behavioural activation, identification and challenge of negative automatic thoughts precede the exploration and discussions of spiritual struggles, gratitude, altruism and spiritual growth.

Similarly, Sabki et al. (2019) revised Ciarrocchi et al.’s (2014) framework and devised a 10 weeks Sharia’a-congruent CBT programme for individuals who present with symptoms of depression and chronic physical illness. Building on Al Ghazali’s (1986) conceptualisation of the human psyche as described above, and on the notion that a person’s character ought to be strengthened through the purification of the soul and the cultivation of divine happiness (Tazkiyah al Nafs Model), Sabki et al. carefully mapped their therapeutic model onto the Muslim scholar’s work.

Specifically, Ghazali (1986) proposed a 5-construct model for the fortification of human nature in which the first step was the development of self-awareness: (i) “Knowledge of Self” (Ma’rifah al Nafs). Individuals were encouraged to find a life purpose that was congruent with the Islamic teachings, to set goals and to identify potential problems before (ii) setting off towards the Purification of the Heart (Takhalli), committing to overcoming negative qualities through understanding the life of the Prophets as described in the Quran and other relevant books. Once the heart was purified, the process of “Cultivation of the Heart” (Tahalli) started (phase iii). The aim of this phase was to build inner strength through remembrance (Zikr) and repentance (tawbah) and to export this renewed energy to the body and the soul. Ongoing Self-Evaluation (phase iv; Muhasabah al Nafs) was deemed to be key to ensure that solutions to problems were found. The ultimate goal was to achieve divine, transcendental Happiness (al Saadah) (phase v) in this world and in the Hereafter.

Linking their 10 weeks programme to the above framework, Sabki et al. (2019) argue that the first 2 sessions (*Building rapport/introduction to Islamically Integrated Cognitive Behavioural Therapy—*IICT and *Walking by Faith*) map onto the first element of Ghazali’s (1986) model. Clients are asked to search within to find that connection with the other and trust the process of therapy, even though it might not initially make sense to them (hence walk by faith). The third session and fourth session, respectively “*the battlefield of the mind”* and *“bringing all thoughts captive”* map onto the “purification of the heart”. Clients are encouraged to identify NATs and their link to emotions. Thought challenges on the basis of religious beliefs and/or contemplative, mindful prayers are introduced as substitutes to secular interventions to encourage individuals to develop more adaptive thinking patterns and/or to stay in the present.

The next four sessions, namely “*dealing with loss”*, “*spiritual struggles”*, “*gratitude”* and “*generosity*” aim at “cultivating the heart” (Ghazali, 1986) through Quran-based cognitive restructuring and discussions of exemplary models of strength and hope. “*Spiritual growth”*, the penultimate session of the Sabki et al. (2019) programme encourages “self-evaluation” (Ghazali, 1986) ahead of “*Hope”,* transcendental Happiness (Ghazali, 1986).

Similarly, Hamdan (2008), Husain and Hodge (2016), and Lodi (2018) suggest modifying CBT’s secular cognitive restructuring statements with more religious ones for clients who might be spiritually inclined. For example: the thought “I had enough/I can’t bear any more” could arguably be challenged with the following extracts from the Quran: “*Allah will not burden a soul with more than it can bear*” (Holy Quran, Al Baqarah, 2:286); “*whoever puts their trust in Allah, he will be enough for them*” (Holy Quran, At Talaq, 65:3) and “*do not lose hope, nor be sad. You will surely be victorious if you are true believers* (Holy Quran, Surat Al-Imran, 3:139). Rothman (2018) further suggests encouraging clients to replace negative thoughts and associated cognitive biases with the practice of “*dhikr*” (remembrance of God), with “*dua’s*” (supplications) or with more helpful religious thoughts, a procedure in line with Seligman’s (2004) positive psychology’s philosophy and with the teachings of Al Balkhi (Badri, 2013).

Likewise, Lodi (2018) recommends reframing secular interventions based on examples from Prophet Mohammed’s life and she suggested enhancing Socratic questioning with a spiritual focus. For example: the classical decentering CBT question “what would my best friend say about this?” could be adapted to reflect a more religious outlook “what would the Prophet Mohammed say/do in these circumstances?”. Further spiritual Socratic questions might allude to what the Quran says about something and how things might affect an individual in the Hereafter (Lodi, 2018). In addition, it has been suggested borrowing elements from third wave CBT and behavioural activation and reframing those within an Islamic paradigm whereby the “compassionate mind” (Gilbert, 2010) is the mind of the prophet Mohammed and behavioural activation is based on prophetically inspired actions (Lodi, 2018).

Mindfulness exercises ought to be adapted to incorporate mindful “Wudu” (ablution), mindful “*Salat*” (praying) and/or mindful “*Tafakkur*” (contemplation of God) (Rothman, 2018; Thomas et al., 2017). These small changes put interventions in line with early Muslim’s writings “*nothing in your prayer counts except that in which you are mindful*” (Al-Ghazali, 2010, p. 68), hence increasing the cultural relevance of the interventions. Using cultural adaptations of secular frameworks is paramount to maximise commitment and engagement with the model (Cucchi et al., 2020) and to protect against the dangers of “hegemonic globalisation”. Indeed, the literature reminds clinicians of the heartfelt cultural disconnection lamented by some Muslims who engaged in secular mindfulness-based interventions: “*most of the stories are not from here, they do not relate to us; we would prefer examples and stories that we could relate to*” (Thomas et al., 2016, p. 301).

Given the above, Acceptance and Commitment Therapy (ACT) metaphor of the “unwelcome party guest” (Oliver, 2011) and mindfulness of emotions exercises ought to be substituted with autochthonous sources. Thomas et al. (2017) uses Jalal al-Din Rumi’s (1997) poem of the “guesthouse” to exemplify the concept of acceptance in Islam. Quotes like “*every morning a new arrival. A joy, a depression, a meanness, (…). Welcome and entertain them all!; the dark thought, the shame, the malice, meet them at the door laughing and invite them in”* (Rumi, 1997—Translation by Coleman Barks) show not only the relevance of CBT-based concepts to Islam, but also its pre-existence to the CBT model.

Whilst early in its development and far from claiming a universally accepted organic theoretical framework, incorporating Islamic principles into CBT seems a useful possibility. Islamically grounded interventions might not be suitable or appealing to everyone, including many Muslims who might prefer secular approaches. At the same time, it is vital to broaden the repertoire of available psychological and psychotherapeutic approaches and offer culturally sensitive frameworks as part of a commitment towards moving away from solely relying on western perspectives when practising and teaching psychology and psychotherapy. The traditional suspiciousness and reluctance that some individuals might experience concerning seeking treatment for mental health difficulties could then be chipped away by presenting an approach that might mirror the client’s belief system closer.

People can be reminded that the Prophet Mohammed explicitly encouraged individuals to “heal themselves” (Asamarai, 2018), “*there is no ailment that Allah has created, except that He also created its remedy*” (Al Bukhari, translation by Khan, 1998). Psychotherapy and psychology can then be presented as two such remedies, which nevertheless require individual efforts and personal agency. Individuals can be reminded that “*Allah will not change the condition of people until they change themselves*” (Holy Quran, Ar-Rad, 13:11), suggesting that personal responsibility and commitment are key. Furthermore, it might be helpful for therapists to develop collaborative relationships with religious leaders. Due to the different idiom of distress in western and non-western cultures as previously discussed, working with religious leaders might, then, help healthcare professionals to develop explanatory models that better capture indigenous beliefs’ systems.

Given that several studies confirm the potential helpfulness of traditional interventions (Nortje et al., 2016; Sorketti et al., 2013; van der Watt et al., 2018), working with religious healers might also help to incorporate mental health services into the community, hence normalising treatment and tackling stigma. However, it is crucial to be clear about the boundaries of psychotherapy and those of religion in such cases. Explicit and transparent discussions about this ought to occur, particularly for those clients who might not be familiar with the process of therapy (York-Al Karam, 2018). Furthermore, far from assuming that all Muslims are monolithic in their approach to religion (Haque, 2018a), integration ought to happen at the individual’s level and following the client’s lead. Indeed, “*there is no compulsion in religion*” (Holy Quran, Al Baqarah, 2: 256).

## Strengths and Limitations

The present paper has both strengths and limitations. As a narrative review, it explores the state-of-the-art literature on the topic of integration of CBT and Islamic values in depth, and offers a bridge between the vast and disjointed array of articles on the subject. Indeed, one of the advantages of narrative reviews is that they present arguments at a theoretical level that empirical studies cannot address, often leading to novel conceptualisations and frameworks (Baumaister & Leary, 1997). This paper achieved these objectives, by integrating and building on the available literature and offering ideas to develop further. At the same time, whilst the topic’s theoretical argument is developed in depth, the exploration of its evidence base is limited. Hence, this review cannot be used as scientific evidence. Furthermore, although the author has endeavoured to present a balanced argument for/against the integration of CBT and Islamic principles, the discussion reflects the author’s interpretation of the literature.

As a result, further theoretical reflections and follow-up studies ought to take place before conclusions are drawn. These reflections should be driven by a bottom-up approach where the targeted population is recognised as the expert and consulted through qualitative studies.

## Conclusions

The impact of globalisation on the conceptualisation and treatment of psychological distress calls for a greater effort to integrate psychological and psychotherapeutic approaches in the respect of indigenous cultures. In light of this, the current paper engaged with the controversy of adapting the CBT approach to incorporate Islamic values. Given that each paradigm is rooted in contrasting ontological and epistemological positions and that acceptance of one model would indirectly influence the therapeutic stance (Fatemi, 2018), it seems crucial for integration to happen at a theoretical level first. Only in this way a psychotherapeutic model would truly connect to people’s needs. Islamic psychotherapy and Islamically integrated approaches have started to emerge (Ciarrocchi et al., York-Al Karam, 2018; Sabki et al., 2019). However, these are in their infancy and lack a systematic approach to their development and a formalised framework. In addition, the few available models mostly borrow from the western tradition. Whilst this might be a valuable tool, the development of a psychotherapeutic approach from the “bottom up” (Sheikh, 2018) is very much overdue.
